# Dual-energy computed tomography has limited sensitivity for non-tophaceous gout: a comparison study with tophaceous gout

**DOI:** 10.1186/s12891-016-0943-9

**Published:** 2016-02-18

**Authors:** Alan N. Baer, Tracie Kurano, Uma J. Thakur, Gaurav K. Thawait, Matthew K. Fuld, Janet W. Maynard, Mara McAdams-DeMarco, Elliot K. Fishman, John A. Carrino

**Affiliations:** Department of Medicine (Rheumatology), Johns Hopkins University School of Medicine, 5200 Eastern Avenue, Suite 4000, Mason Lord Center Tower, Baltimore, MD 21224 USA; Russell H. Morgan Department of Radiology and Radiological Science, Johns Hopkins University School of Medicine, Baltimore, MD USA; Siemens Medical Solutions USA Inc., Malvern, PA USA; Department of Epidemiology, Johns Hopkins Bloomberg School of Public Health, Baltimore, MD USA

**Keywords:** Gout, Dual-energy computed tomography, Imaging, Diagnosis, Tophaceous gout, Non-tophaceous gout

## Abstract

**Background:**

Dual-energy computed tomography (DECT) is a new diagnostic tool for gout, but its sensitivity has not been established. Our goal was to assess the sensitivity of DECT for the detection of monosodium urate (MSU) deposits in non-tophaceous and tophaceous gout, both at the level of the patient and that of the individual joint or lesion.

**Methods:**

DECT was performed on 11 patients with crystal-proven non-tophaceous gout and 10 with tophaceous gout and included both the upper and lower extremities in 20/21 patients. DECT images were simultaneously acquired at 80 and 140 kV and then processed on a workstation with proprietary software using a two-material decomposition algorithm. MSU deposits were color coded as green by the software and fused onto grey-scale CT images. The number and location of these deposits was tallied independently by two DECT-trained radiologists blinded to the clinical characteristics of the patient. Sensitivity of DECT was defined as the proportion of patients with a confirmed diagnosis of gout which was correctly identified as such by the imaging technique. All patients provided informed consent to participate in this IRB-approved study.

**Results:**

MSU deposits were detected by DECT in ≥1 joint area in 7/11 (64 %) patients with non-tophaceous gout, but were only detected in 3/12 (25 %) joints proven by aspiration to be affected with gout. Inclusion of the upper extremity joints in the scanning protocol did not improve sensitivity. All 10 patients with tophaceous gout had MSU deposits evident by DECT. The sensitivity of DECT for individual gouty erosions was assessed in 3 patients with extensive foot involvement. MSU deposits were detected by DECT within or immediately adjacent to 13/26 (50 %) erosions.

**Conclusions:**

A DECT protocol that includes all lower extremity joints has moderate sensitivity in non-tophaceous and high sensitivity in tophaceous gout. However, DECT has lower sensitivity when restricted to individual crystal-proven gouty joints in non-tophaceous disease or individual erosive lesions in tophaceous gout. The detection of MSU deposits by DECT relates to their size and density and the detection parameters of the DECT scanner and adjustment of the latter might improve sensitivity.

## Background

Gout is an inflammatory arthritis characterized by the deposition of monosodium urate (MSU) crystals in joints, cartilage, and soft tissues [1]. The untreated disease can lead to formation of tophi, chronic synovitis, and joint damage [1, 2]. Thus, the establishment of a diagnosis and initiation of therapy early in disease are essential to the maintenance of normal function and quality of life. Demonstrating MSU crystals in the joint fluid or in a tophus is the gold standard for gout diagnosis. However, many health care providers do not perform arthrocenteses and the identification of MSU crystals can be challenging, especially in early disease. In the absence of MSU crystal demonstration in joint or tophus aspirates, clinical, radiographic, and laboratory criteria can be helpful in diagnosis. However, characteristic radiographic findings are only seen late in the disease, including “punched-out” erosions with overhanging edges and sclerotic margins, often in association with asymmetric soft tissue masses [3, 4]. Various advanced imaging techniques are being utilized, including ultrasonography with power Doppler, magnetic resonance imaging (MRI), and conventional computed tomography (CT). Each has their unique advantages and disadvantages. However, none are specific enough to facilitate or confirm a diagnosis of gout [5]. Dual-energy CT (DECT) is a new technique that allows identification of MSU crystal deposits. With this imaging methodology, the compositions of different tissues are determined by analyzing the difference in attenuation in a material exposed simultaneously to two different X-ray spectra. It has high accuracy and sensitivity for the identification of MSU and calcium kidney stones [6]. Whereas its sensitivity has been reported to be 100 % for clinically-overt tophaceous disease [7–9], its sensitivity has not been fully established for non-tophaceous disease where its diagnostic utility would be the greatest.

Our goal was to assess the sensitivity of DECT for the detection of monosodium urate (MSU) deposits in non-tophaceous and tophaceous gout, both at the level of the patient and that of the individual joint or lesion.

## Methods

### Study patients

We studied 21 patients with gout as defined by the American College of Rheumatology preliminary criteria [10]. With one exception, all patients were recruited from the clinical practice of one of the authors (ANB) and underwent a comprehensive musculoskeletal examination at the time of recruitment. The patients included 1) 11 with non-tophaceous gout, defined by the demonstration of MSU crystals on a joint aspirate [performed in 10 of the 11 patients by one of the authors (ANB)] and the absence of tophi on physical examination or erosions on available radiographs and 2) 10 with tophaceous gout, defined by one or more of the following: the presence of palpable tophi (*n* = 5), the presence of characteristic radiographic erosions (*n* = 7), or gross MSU deposits in a surgical specimen (*n* = 3).

The Johns Hopkins Medicine Institutional Review Boards approved the study. All patients provided written informed consent before inclusion in the study.

### Study protocol

Each patient underwent DECT of their hands, wrists, elbows, knees, ankles, and feet using the imaging protocol described below. The initiation of urate-lowering therapy (ULT) was not withheld in treatment-naïve patients, although the scan was completed as soon as possible after ULT was initiated. Each patient underwent a comprehensive musculoskeletal examination. Clinical data obtained for this study included demographics (age, gender, ethnicity), gout history (duration of disease, therapeutic interventions, frequency of gout flares), arthrocentesis if the disease was not previously crystal-proven, radiographs performed as part of the clinical assessment, and laboratory evaluation including serum urate levels.

### Dual-energy computed tomography protocol

DECT images were obtained of the four limbs scanned in pairs: the hands/wrists, feet/ankles, elbows, and knees. With each scan, the patient was positioned so that the relevant joints were in a central location within the dual energy field. Each joint area had a scan field which ensured that all articulations were visualized in full and included a minimum of 4 cm of adjacent extremity for the elbow, knees, wrists, and ankles. Scans were performed using a dual-source DECT scanner (SOMATOM Definition Flash Dual-Source CT scanner; Siemens Healthcare, Forchheim, Germany). Parameters were 80/Sn140 kVp and 211 mAs for tube A with tube B automatically adjusted to maintain a 2:1 ratio. Collimation of 0.6 mm was reconstructed to yield a 0.75-mm slice thickness. A material decomposition algorithm was performed on a multi-modality workspace (SW-Version VE52 Siemens Healthcare) using Siemens syngo.via dual-energy software package (SW-Version VA20). The material-specific difference in attenuation of urate between the two energy levels at 80- and Sn140-kV allowed accurate detection of MSU, which was then color coded as green and fused onto the standard greyscale CT image. These could be reviewed as both 2D cross-sectional and 3D surface rendered images. The threshold ratio parameter was set at 1.36, with a range of Hounsfeld units of 150 – 500 HU.

Each DECT scan was read by two of three musculoskeletal or CT radiologists (EKF, JAC, or UT, with 32, 16, and 1 years of experience, respectively) and scored with respect to the presence and number of MSU deposits at each joint. For cases in which a discrepancy existed, agreement was reached by consensus. These radiologists were blinded to the clinical characteristics of the patients and trained to exclude known artifacts, including those related to nailbeds, skin, metal prostheses, and beam hardening, from their analyses [11].

### Statistical analysis

Descriptive statistics were used to characterize the baseline features of the cohort. Proportions were used to summarize categorical variables and median [range] or mean [standard deviation] for continuous variables. The Wilcoxon rank-sum test was used to compare continuous variables and the two-sided Fisher exact test was used to compare categorical variables. Correlations between continuous variables were tested using the Spearman rank correlation coefficient. Sensitivity of DECT was defined as the proportion of patients with a confirmed diagnosis of gout (or specific joint involved by gout) which was correctly identified as such by the imaging technique. *P* values less than 0.05 were considered statistically significant. Analyses were conducted using JMP (Version 11, SAS Institute Inc., Cary, NC).

## Results

### Demographic and clinical characteristics

The demographic and clinical characteristics of the patients at the time of DECT are summarized in Table 1. The study patients included 17 men and four women with a median age of 59 years (range, 43–89 years). There was no significant difference in age between the patients with non-tophaceous and tophaceous gout. The median duration of gout was 0.8 years (range 0.2-13 years) for the non-tophaceous gout patients and 10.2 years (range 1–28 years) for those with tophaceous gout. Among the 11 non-tophaceous gout patients, seven had had gout for less than one year and nine had had more than one gout attack. The maximum recorded serum urate value was higher for the tophaceous than the non-tophaceous gout patients, a difference that did not reach statistical significance (median value, 594.8 vs 529.4 μmol/L, *p* = 0.18).

**Table 1:** Characteristics of non-tophaceous and tophaceous gout patient groups

	Non-tophaceous	Tophaceous
Number of patients	11	10
Age, years; median (range)	64 (43–72)	56 (49–89)
Male; number (%)	9 (82)	8 (80)
Caucasian; number (%)	9 (82)	8 (80)
Duration of gout, years; median (range)	0.8 (0.2-13)	10.2 (1–28)
Glomerular filtration rate <60 ml/min; number (%)	2 (18)	5 (50)
BMI, kg/m^2^; mean ± SD	29.8 ± 4.0	30.0 ± 3.2
Maximum serum urate (μmol/L), median (range)	529.4 (339.0-814.9)	594.8 (458.0-850.6)
Urate-lowering therapy at time of scan, number (%)	9 (82)	7 (70)

Among the 11 non-tophaceous gout patients, MSU crystals were demonstrated in 12 joint aspirates, including those of the 1^st^ metatarsophalangeal (*n* = 7), knee (*n* = 2), ankle (*n* = 2), and metacarpophalangeal (*n* = 1) joints (Table 2). The median interval between these joint aspirations and DECT was 37 days (range, 21 – 149). At the time of DECT, nine of these 11 patients were on ULT with a median duration of 41 days (range 6–126 days). Serum urate values obtained within 30 days of DECT were < 6 mg/dl in three of eight patients.

**Table 2:** Results of DECT scanning in patients with non-tophaceous gout

Patient	Distribution of MSU deposits detected by DECT scanning		
	Any joint	Joint shown to have MSU crystals by aspiration	
1	-	-	First MTP
2	+	-	First MTP
3	-	-	First MTP
4	+	+	Knee
5	-	-	Ankle
6	+	-	First MTP
7	+	-	First MTP
8	+	+	Knee
9	-	-	First MTP
10	+	+	ankle
		-	1^st^ MTP
11	+	-	MCP
Overall sensitivity	7/11 (64 %)	3/12 (25 %)	

Among the 10 tophaceous gout patients, five had palpable tophi, three with associated radiographic erosions and one with tophaceous material in a surgical specimen (Table 3). The remaining five patients had tophaceous material in a surgical specimen (*n* = 2) or characteristic erosions on radiographs (*n* = 4). Seven of the 10 patients with tophaceous gout had had their diagnosis confirmed by microscopic demonstration of MSU crystals.

**Table 3:** Results of DECT scanning in patients with tophaceous gout

Patient	Crystal-proven diagnosis	MSU deposits evident by DECT	Correlation of clinical and radiographic findings with DECT imaging for MSU deposits		
			Evidence for tophaceous disease	Site of tophi	DECT MSU deposit at site
12	No	Yes	Erosions on radiograph	Left 1^st^ MTP	+
13	Yes	Yes	Surgical specimen	Left wrist	+
			Erosions on radiograph	Right 2^nd^ MTP	+
14	No	Yes	Erosions on radiograph	Right 1^st^ MTP	-
15	Yes	Yes	Palpable tophi	Right olecranon bursa Left olecranon bursa	+ +
			Erosions on radiograph	Right 1^st^ MTP	+
16	Yes	Yes	Surgical specimen	Right peroneus tendon	-
17	Yes	Yes	Palpable tophi	Dorsum right long finger PIP Dorsum right little finger PIP	Upper extremity DECT not done
18	Yes	Yes	Erosions on radiograph	Right carpus Left ring finger PIP	- -
19	No	Yes	Palpable tophi	Right Olecranon bursa Left olecranon bursa Left ulnar styloid Dorsum left ring finger MCP Right thumb pad Volar left little finger PIP Right index fingertip pad Right 1^st^ MTP Left 1^st^ MTP	- + - - + - + + +
20	Yes	Yes	Palpable tophi	Left thumb MP Left little MCP Left index PIP Left long PIP radial aspect Left ring PIP Left little between PIP/DIP dorsal Right index PIP Right little DIP Right long PIP Dorsum left foot Dorsolateral left ankle Lateral left midfoot Right 1^st^ MTP Right 2^nd^ DIP (draining)	+ + + + - + + - + + + + + +
			Erosions on radiograph	Right carpus Right index PIP Right little DIP	+ + +
21	Yes	Yes	Surgical specimen	Left 1^st^ MTP	+
			Erosions on radiograph	Left 1^st^ MTP	+
			Palpable tophus	Left 1^st^ MTP	+

### DECT scanning in non-tophaceous gout

Among the 11 patients with non-tophaceous gout, seven (64 %) had MSU deposits detected by DECT of both upper and lower extremity joints (Table 2). The MSU deposits were present in only three of the 12 joints (25 %) shown to have MSU crystals by polarized light microscopic analysis of aspirated material. MSU deposits were present in the elbows of three patients, but in each of these they were also found in one or more lower extremity joints.

In a comparison of the patients with and without MSU deposits by DECT, the median age of the seven patients with deposits was 57 years and that of the four patients without deposits was 66 years (*p* = 0.07, Wilcoxon rank sum test). There were no significant differences in duration of clinical gout or maximum serum urate value between the two groups. Among the nine patients on ULT at the time of the scan, there was no significant difference in the duration of this therapy between the seven with and the two patients without MSU deposits.

The number of MSU deposits ranged from 1–15 (median 3) among the seven patients with positive DECT. The number of these deposits did not correlate significantly with the maximum serum urate level.

### DECT scanning in tophaceous gout

**Fig. 1:**
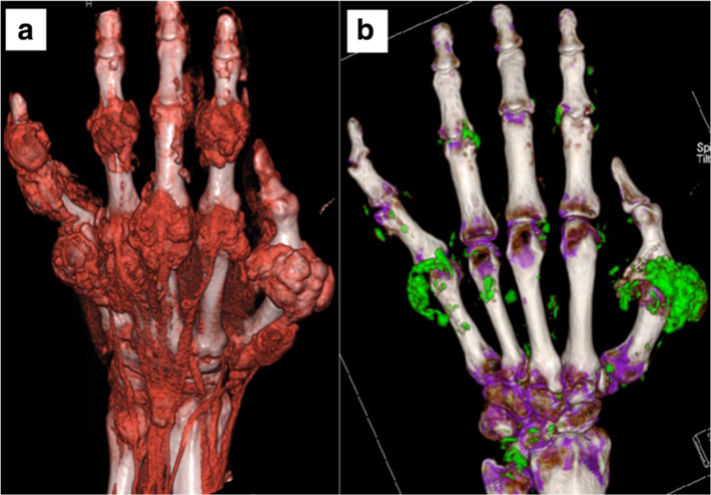
Patient 20. Large tophi are evident in the fingers with 3D volume rendering of the 2-D CT images, using proprietary software for the digital reconstruction and application of colors and varying degrees of transparency to the tissues panel (**a**). With DECT scanning panel (**b**), MSU deposits (evident by their green coding) conform to the areas of tophaceous deposits, but are sparse in some affected areas and absent in others (e.g. little finger DIP and long finger PIP)

MSU deposits were evident by DECT in each of the 10 patients (100 %) with tophaceous gout (Table 3). We sought to determine the correlation between MSU deposits on DECT with tophi evident by clinical examination or by the presence of characteristic erosions on radiographs or CT images. Of the five patients with palpable tophi, a correlative analysis could be performed on three (one patient had his solitary tophus surgically excised and the other had DECT of only the lower extremities). In these three patients (numbers 15, 19, 20), there were 25 discrete palpable tophi. Corresponding MSU deposits were detected with DECT in 19 (76 %). An example of this is shown in Fig. 1. This patient had soft tissue masses around the metacarpophalangeal, proximal interphalangeal and distal interphalangeal joints of his hand, evident both by palpation and by 3D-volume rendering of the CT images. MSU deposits evident by DECT largely conformed to these soft tissue masses, but were sparse in some affected joints and absent in others (e.g. little finger DIP and long finger PIP joints).

**Fig. 2:**
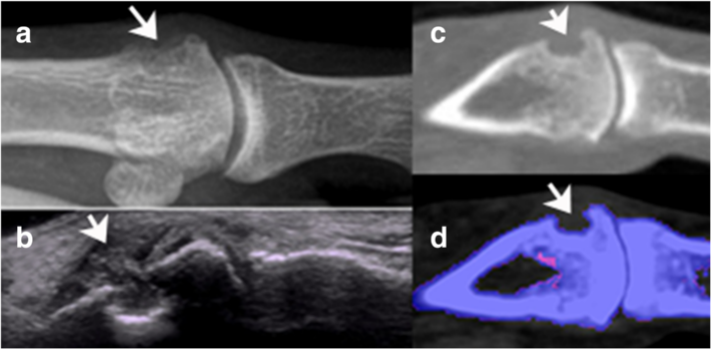
Patient 14. Erosion of distal right metatarsal head, imaged by radiograph (panel (**a**), arrow), ultrasound (panel (**b**), arrow), CT (panel (**c**), arrow) and DECT (panel (**d**), arrow). Fluffy, hyperechoic material is evident in the synovial space on ultrasound (panel (**b**), arrow). Note the absence of any green pixellated areas (MSU deposits) in panel (**d**). The DECT scan was positive for MSU deposits in the left distal metatarsal head (not shown)

Ten discrete osseous erosions were evident on radiographs obtained on affected joints of seven patients prior to DECT. MSU deposits were evident at the site of seven of these erosions. An example of metatarsal head erosion not associated with MSU deposits by DECT is shown in Fig. 2. In this patient, ultrasonography demonstrated fluffy hyperechoic material in the synovial space, suggestive of tophaceous material.

**Fig. 3:**
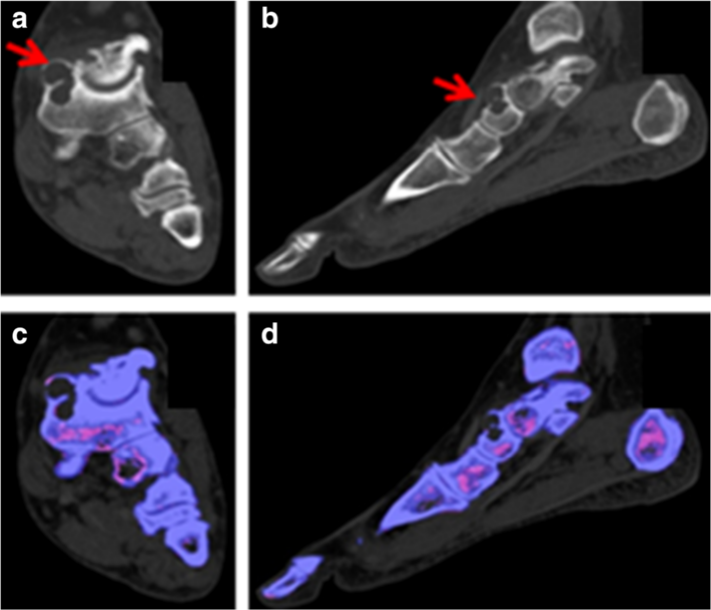
Patient 15. There is a large erosion of the dorsal navicular bone, seen on the coronal and sagittal CT images (panels (**a**) and (**b**), red arrows) but no corresponding green-coded (MSU) deposits in or around the erosion on the DECT images (panels (**c**-**d**))

Three patients had extensive erosions in their feet, evident by CT and defined by a well-defined cortical break and overhanging edges. At the time of DECT scan, two had initiated urate-lowering therapy 34–100 days before the scan, but none of the three had a serum urate level <6.8 mg/dl. By fusing the dual-energy processing with the CT images, we tabulated the number of erosions with and without corresponding MSU deposits in the erosion cavity or in the surrounding soft tissue mass. Among these three patients, there were 26 erosions, only 13 (50 %) of which had associated MSU deposits. This failure to detect MSU deposits could not be overcome by changing the lower limit of the density threshold of the DECT from 150 to 135 Hounsfield units. An example of a large erosion evident by CT and lacking associated MSU deposits is shown in Fig. 3.

## Discussion

The diagnostic utility of DECT in the evaluation of arthritis hinges on its ability to detect MSU deposits in patients with various stages of gout. In the current study, we compared the sensitivity of DECT in patients with crystal-proven, non-tophaceous gout and those with tophaceous gout, with or without radiographic erosions. In our 11 patients with non-tophaceous gout, DECT of joints in all four extremities demonstrated MSU deposits in seven, equating to an overall sensitivity of 64 %. Importantly, these deposits were only detected in three of 12 (25 %) joints shown to have MSU deposits by aspiration and polarized light microscopic analysis of the synovial fluid or soft tissue material. In this small series of seven non-tophaceous gout patients with MSU deposits detected by DECT, inclusion of the upper extremities in the scanning protocol served to identify MSU deposits in the elbows of three, but each had concomitant deposits in their lower extremities. Thus the sensitivity of DECT was not increased by the inclusion of the upper extremities but was increased by inclusion of all lower extremity joints.

Our protocol for DECT of both the upper and lower extremities identified MSU deposits in each of our 10 patients with tophaceous gout. This 100 % sensitivity of DECT for tophaceous gout is consistent with three other studies [7–9]. However, this study highlights the limitations of DECT in identifying tophaceous deposits. Specifically, there were notable examples where MSU deposits could not be detected in areas of tophaceous deposits, either judged clinically by physical examination or by radiographic, ultrasonographic, or CT imaging. This failure to detect MSU deposits in sites of tophi could not be overcome by a change in detection thresholds of the DECT imaging software. Thus, there may be limitations in the sensitivity of DECT for defining gout as the etiology for individual erosions or periarticular soft tissue masses.

The sensitivity of DECT in crystal-proven, non-tophaceous gout has been evaluated in three other studies, with reported sensitivities ranging from 79–90 % [12–14]. In these studies, DECT was restricted to the feet [13], the clinically affected joint [12], or the most prominently involved joint regions [14]. The sensitivity of DECT for the diagnosis of non-tophaceous gout was lower in our series of patients, particularly when judged by the detection of MSU deposits in the joints from which MSU crystals were detected by joint aspiration.

There are several potential reasons for the lower sensitivity noted in our study. First, we may have included patients with a shorter duration of gout or a lesser degree of hyperuricemia, leading to MSU crystal deposition below the threshold of detection of DECT. The reported sensitivity of DECT for the detection of MSU deposits is proportional to the stage of gout, being 0.03-24 % in asymptomatic hyperuricemia [13, 15], 79–80 % in patients with short-duration acute gout [12, 13], 84 % in long-duration (≥3 years) acute gout [13] and 100 % in tophaceous gout [7–9]. The limit of detection of DECT is generally considered to be 2 mm, so microscopic tophi may be missed [16]. The mean duration of gout was shorter in our subjects compared to the participants of two other studies where these data are provided (3.1 years in our study compared to 3.8-11 years in the other studies) [13, 14]. In the study of Manger et al., only two of four patients presenting with their first gout flare had DECT evidence of MSU deposition. The mean serum urate values were comparable in our subjects to those of the other three studies, so an effect of hyperuricemia severity could not be assessed. Second, our study protocol allowed patients to initiate ULT before DECT. Nine of our 11 subjects with non-tophaceous gout were on ULT for a median of 35 days (mean 1.5 months). Eight of these had a serum urate measured within 30 days of DECT and all but three had a serum urate <6 mg/dl. However, 82 % of subjects in the study of Dalbeth et al. were on ULT for an average of 34 months [13]. Similarly, MSU deposits were detected in 37 of 40 gout patients studied by Choi et al., despite the fact that 35 had been treated with ULT [17]. The rate of dissolution of MSU deposits has been shown to be slow in patients on appropriately-dosed xanthine oxidase inhibitor or uricosuric therapy, as measured by clinical exam [18] and by DECT [9, 19]. The size of subcutaneous tophi, measured with calipers, did not change significantly over a one-year period in a clinical trial comparing febuxostat and allopurinol, despite normalization of the serum urate [20]. Third, our imaging parameters may have been different from those of other investigators, although we used parameters established by the manufacturer as optimal for gout imaging.

The failure of DECT to detect MSU crystal deposits at sites of tophi evident by physical or pathologic examination or other imaging methods has been noted by other investigators. Although DECT identified four times more urate deposits than physical examination in the study of Choi et al., there were two patients in whom palpable tophi did not have a corresponding MSU deposit by DECT [8]. Glazebrook et al. reported a patient with an inflamed wrist, small erosions at the head of the third metacarpal, and negative DECT but evidence of MSU crystals by joint aspiration and synovial biopsy [16]. In a study correlating structural joint damage on radiography of the feet with DECT MSU crystal deposition, Dalbeth et al. found MSU deposits in 112/262 (43 %) joints with radiographic erosions. The prevalence of these deposits in individual joints correlated positively with the joint erosion score [7]. In 161 gout patients studied by Hu et al., MSU deposits were evident by DECT in 121. Among the 40 with negative DECT, eight had MSU deposits evident by conventional CT [21]. Similarly, McQueen detected tophi by MR but not by DECT in 10/150 joint sites in a study of 10 tophaceous gout patients [22]. In the study of Huppertz et al. comparing DECT and ultrasound for the diagnosis of gout in 39 patients, MSU deposits were detected by US but not by DECT in three patients [23]. At the level of individual joints, the disparity was more striking. As an example, MSU deposits were evident by DECT in 26 % and by ultrasound in 74 % of the 78 first metatarsophalangeal joints [23]. Finally, Melzer et al. reported a patient with tophaceous gout who underwent DECT of her feet one week before her death [24]. Two large soft tissue tophi, one around the ventral ankle joint and the other at the distal left great toe, had minimal to no green-coded images on DECT. At post-mortem examination, both of these soft tissue masses were confirmed to be tophi with a central crystalline core, the latter measuring at least 1 cm in one dimension in each.

There are several explanations for the occasional failure of DECT to detect MSU deposits at sites of tophi. Dalbeth et al. noted a linear relationship between erosion size and MSU crystal deposition evident by DECT, suggesting that the resolution of DECT may not be sufficient to detect small tophi [7]. This issue would apply in particular to MSU deposits detected by ultrasound but not by DECT. The parameter ratio for DECT is set by the manufacturer to optimize differentiation of urate- and calcium-containing voxels. However, several investigators have shown that the use of a different parameter ratio can correct previously false-negative DECT scans for MSU deposits detected by conventional CT [21] or MR imaging [22]. Finally, DECT may only detect dense tophi. Lockmann et al. showed that DECT did not detect MSU crystals in a sample of white synovial fluid containing a high concentration of these crystals by polarized light microscopy (i.e. “gout milk”) [25]. In the pathologic examination conducted by Melzer et al., tophi with a density throughout most of their volume below 150 Hounsfield units were not color-coded by the DECT imaging software, thereby limiting the sensitivity of DECT for low-density tophi [24].

Our study was limited by the absence of control subjects to assess specificity of DECT. The small size of our study did not allow determination of the sensitivity of DECT for the entire spectrum of non-tophaceous gout, but served to illustrate the limitations of the technique in patients with early disease whose diagnosis was confirmed by joint aspiration.

## Conclusions

In conclusion, a DECT scanning protocol that includes all lower extremity joints has moderate sensitivity in non-tophaceous and high sensitivity in tophaceous gout. However, DECT has lower sensitivity when restricted to individual crystal-proven gouty joints in non-tophaceous disease or individual erosive or tophaceous lesions in tophaceous gout. The detection of MSU deposits by DECT depends on their size and density and the detection parameters of the DECT scanner. The latter could potentially be improved with an adjustment of the parameters used to both acquire and then process the DECT images.

### Ethics approval and consent to participate

The Johns Hopkins Medicine Institutional Review Boards approved the study (NA00067327). All patients provided written informed consent before inclusion in the study.

## Abbreviations

DECT: Dual-energy computed tomography
CT: Computed tomography
MSU: Monosodium urate
ULT: Urate-lowering therapy
MTP: Metatarsophalangeal
MCP: Metacarpophalangeal
DIP: Distal interphalangeal
PIP: Proximal interphalangeal
BMI: Body mass index
